# Performances of Anion-Exchange Blend Membranes on Vanadium Redox Flow Batteries

**DOI:** 10.3390/membranes9020031

**Published:** 2019-02-17

**Authors:** Hyeongrae Cho, Henning M. Krieg, Jochen A. Kerres

**Affiliations:** 1 Institute of Chemical Process Engineering, University of Stuttgart, 70199 Stuttgart, Germany; hyeongrae.cho@icvt.uni-stuttgart.de; 2 Faculty of Natural Science, North-West University, Focus Area: Chemical Resource Beneficiation, Potchefstroom 2520, South Africa; henning.krieg@nwu.ac.za

**Keywords:** vanadium redox flow battery, anion-exchange blend membrane, F6-PBI, bromomethylated PPO, 1,2,4,5-tetramethylimidazole

## Abstract

Anion exchange blend membranes (AEBMs) were prepared for use in Vanadium Redox Flow Batteries (VRFBs). These AEBMs consisted of 3 polymer components. Firstly, PBI-OO (nonfluorinated PBI) or F6-PBI (partially fluorinated PBI) were used as a matrix polymer. The second polymer, a bromomethylated PPO, was quaternized with 1,2,4,5-tetramethylimidazole (TMIm) which provided the anion exchange sites. Thirdly, a partially fluorinated polyether or a non-fluorinated poly (ether sulfone) was used as an ionical cross-linker. While the AEBMs were prepared with different combinations of the blend polymers, the same weight ratios of the three components were used. The AEBMs showed similar membrane properties such as ion exchange capacity, dimensional stability and thermal stability. For the VRFB application, comparable or better energy efficiencies were obtained when using the AEBMs compared to the commercial membranes included in this study, that is, Nafion (cation exchange membrane) and FAP 450 (anion exchange membrane). One of the blend membranes showed no capacity decay during a charge-discharge cycles test for 550 cycles run at 40 mA/cm^2^ indicating superior performance compared to the commercial membranes tested.

## 1. Introduction

Due to the environmental impact of using fossil fuels and their resources, producing carbon dioxide which is considered the main contributor to global warming, the use of renewable sources for generating electric energy is attracting increasing scientific interest [1]. Developing alternative sources however not only entails the development of energy generating but also energy storage capacity. Among the energy storage systems which are currently available, the redox flow battery (RFB) is thought of as one of the most promising candidates because of its storage capacity only being dependent on size of the electrolyte tanks, design flexibility and safety [2]. Among the suggested RFBs currently available, the all vanadium redox flow battery (VRFB) which was first proposed and developed by Skyllas-Kazacos and co-workers in 1985 [3], is the most extensively studied, advanced and only commercially available and widely distributed RFB to date [4]. In VRFBs, ions of vanadium (V) with different oxidation states are used as both electrolytes (V(+2)/V(+3) as the negative electrolyte and V(+4)/V(+5) as the positive electrolyte). This avoids the problem of cross-contamination even though crossover can take place resulting in self-discharge [5]. In addition to the general advantages of RFBs, VRFBs have the additional advantages of room temperature operation, relatively rapid response times and long cycle times [6]. In VRFBs, the membrane is one of the more important components acting as a separator between the two electrolytes used, where it not only prevents the crossover of the electrolytes between the positive and negative half cells but also allows the permeation of charge balancing ions such as H^+^, SO_4_^2−^ and HSO_4_^−^ to complete the electrical circuit. Accordingly, such membranes should have high ionic conductivity and chemical stability during operation at an affordable price especially when considering larger scale commercial applications [7]. Both cation exchange membranes (CEMs) and anion exchange membranes (AEMs) can be used in VRFBs. For AEMs, the positively charged cationic fixed ions repel the positively charged vanadium ions—which is known as the Donnan repelling effect—resulting in a reduction of the vanadium ion crossover [8]. A general disadvantage is that AEMs generally have lower conductivities than CEMs [9]. However, upon immersion of AEMs in the electrolyte solutions, additional sulfuric acid can be absorbed leading to improved conductivities [10]. As a result, AEMs are intensively studied in terms of their suitability in VRFBs. Recent studies, where for example quaternized Diels-Alder poly(phenylene) membranes were developed and tested, showed a higher capacity retention during a cycling test than Nafion [11]. In another study, a quaternary ammonium functionalized Radel (QA-Radel) membrane with an ion exchange capacity of 2.0 mmol/g displayed better coulombic efficiency and capacity retention than Nafion when used in a VRFB [12]. Similarly, quaternized poly (phthalazinone ether ketone ketone) membranes when used in VRFBs showed an almost 100% coulombic efficiency measured by a charge-discharge cycle test for 100 cycles at a current density of 80 mA/cm^2^ [13]. Recently, polysulfone-based crosslinked AEMs with a diamine-based crosslinker were developed and applied in VRFBs [14]. One of those membranes showed a coulombic efficiency of 100% during 100 charge-discharge cycles measured at a current density of 50 mA/cm^2^.

Using acid-base blending to prepare membranes has led to improved membrane properties such as mechanical, thermal and chemical stability as well as their performances in VRFBs [15]. Accordingly, an amphoteric blend membrane consisting of sulfonated poly(ether ether ketone) and quaternized poly(ether imide) showed higher coulombic and energy efficiencies without significant declines, compared to a pristine sulfonated poly(ether ether ketone) as well as Nafion membranes for 100 cycles measured at 50 mA/cm^2^ [16]. Similarly, acid-base blend membranes from imidazolium-functionalized polysulfone blended with sulfonated poly(ether ether ketone) exhibited an improved performance than a Nafion membrane in terms of coulombic and energy efficiencies as well as during a self-discharge test [17]. These studies have clearly shown that acid-base blend membranes have the potential to improve further on existing VRFB performance. When using acid-base blending, literature has shown that 3-component blend membranes perform better in VRFBs than 2-component blend membranes [18]. Accordingly, in this study, anion exchange blend membranes (AEBMs) composed of 3 blend components were prepared and characterized in a VRFB.

It is known that the binding energy of a C-F bond is higher than that of a C-H bond [19]. As a result, partially fluorinated polymer blend membranes in VRFB operation have a higher chemical stability in terms of molecular weight changes during change/discharge cycles [15]. To confirm this, 3-component AEBMs were prepared and applied to VRFBs using different combinations of blend polymers. In this study, two polybenzimidazoles (nonfluorinated PBI-OO or partially fluorinated F6-PBI) were used as the matrix polymer to provide mechanical strength. Bromomethylated PPO was used as the anion-exchange precursor that was quaternized with 1,2,4,5-tetramethylimidazole (TMIm). Finally, two sulfonated polymers (a partially fluorinated polyether or a non-fluorinated poly (ether sulfone)) were investigated as ionic macromolecular crosslinkers. Finally, the efficiency of the partially fluorinated and non-fluorinated AEBMs was determined in a VRFB.

## 2. Materials and Methods

### 2.1. Materials

**Figure 1:**
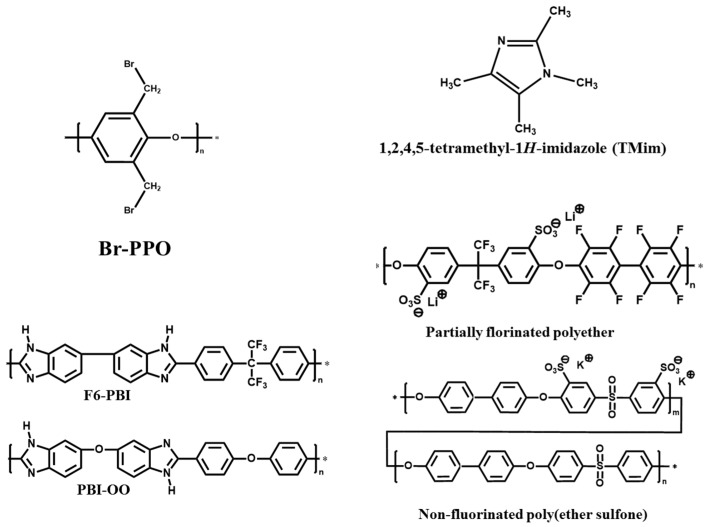
Structures of polymers and TMIm used in this study.

All chemicals were used as received without further purification. Poly(2,6-dimethyl-1,4-phenylene oxide) (PPO) was purchased from Sigma Aldrich and Bromination of PPO was conducted as described in literature [20]. Poly[(1-(4,4′-diphenylether)-5-oxybenzimidazole)-benzimidazole] (PBI-OO) was obtained from FuMA-Tech GmbH, Germany. Fluorinated PBI (F6-PBI) was purchased from Yanjin Technology, Shenzhen, China. The sulfonated polymers were prepared as previously described in the literature [21]. 1,2,4,5-Tetramethylimidazole (TMIm) was purchased from TCI Chemicals. *N*,*N*-Dimethylacetamide (DMAc) and methanol were purchased from VWR International GmbH, Bruchsal, Germany. Potassium carbonate was purchased from ABCR GmbH, Karlsruhe, Germany. Sulfuric acid, potassium hydroxide, 0.1 N standard hydrochloric acid, sodium chloride and sodium hydroxide were purchased from Carl Roth GmbH, Karlsruhe, Germany. The vanadium electrolyte solution (1.6 M vanadium in 30% sulfuric acid: 50% VO^2+^ and 50% V^3+^), was provided by RIVA GmbH Batteries, Backnang, Germany. The structures of the polymers components used in this study are presented in Figure 1. Reference membranes Nafion^®^212 (N212) and Fumasep^®^FAP-450 (FAP 450) were supplied by Ion Power GmbH (München, Germany) and Fumatech GmbH (Ludwigsburg, Germany), respectively.

### 2.2. Membranes Preparation

**Figure 2:**
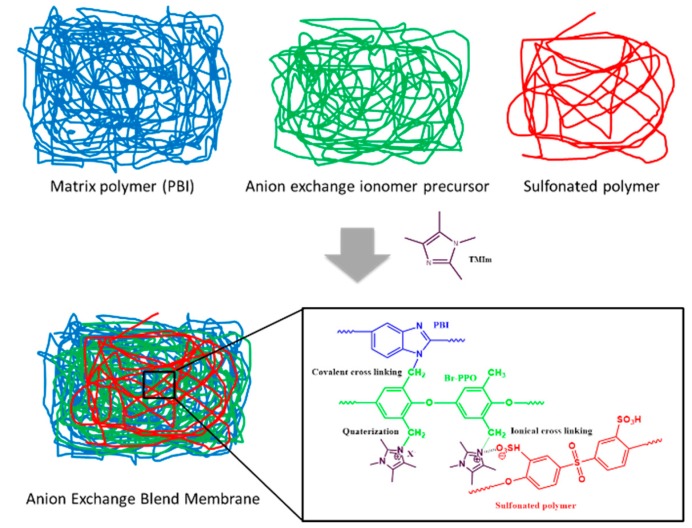
A diagrammatic depiction of the preparation of the anion-exchange blend membranes.

A diagrammatic depiction of how the anion-exchange blend membranes were prepared, is shown in Figure 2. Initially, each polymer solution except for F6-PBI (F6-PBI: 5 wt % in DMAc) was prepared separately as a 10 wt % solution in DMAc. After mixing the polymer solutions in specific ratios, TMIm (same equivalent to the Br-PPO) was directly added to the polymer solution and homogenized. When using F6-PBI during membrane preparation, additional DMAc was added due to the high viscosity of the 5% F6-PBI solution. The blend polymer solution was then cast onto a glass plate, followed by solvent evaporation in a convection oven (pre-heated at 40 °C) at 60 °C for 24 h. After solvent evaporation, the membrane was removed from the glass plate by soaking in water. Subsequently, the membranes were soaked in 1 M sulfuric acid for 24 h at an ambient temperature with one replacement of the 1 M sulfuric acid solution during this period. The membranes were washed with deionized water several times at room temperature to remove all excess sulfuric acid and kept in plastic zipper bags.

### 2.3. Membranes Characterization

#### 2.3.1. Ion Exchange Capacity (IEC)

The membranes were immersed in a 1 M KOH solution for 1 day at 90 °C to convert the bromide to the hydroxide form. After ion exchange, the membranes were washed with water intensively to remove excess KOH on the membrane surface. Subsequently, the membranes were soaked in a 60 mL saturated NaCl solution in order to convert the hydroxide to chloride for 1 day. Subsequently, 3 mL of a standard 0.1 N HCl solution was added to the saturated sodium chloride solution and kept overnight. Then, the membranes were washed with 25 mL distilled water and this water was transferred to the saturated sodium chloride solution. The back titration was carried out with a 0.1 N NaOH solution. Finally, the membranes were thoroughly washed with water and dried at 60 °C. The total IEC was calculated by using Equation (1).

(1) $$IEC=\frac{C_{HCl}\times \;V_{HCl}\;-\;C_{NaOH}\;\times \;V_{NaOH}}{m_{dry}}$$

where IEC is the ion exchange capacity (OH form, mmol/g), C_HCl_ is the concentration of a HCl solution (mmol/ml), V_HCl_ is the used volume of a HCl solution (ml), C_NaOH_ is the concentration of a NaOH solution (mmol/ml), V_NaOH_ is the added volume of a NaOH (ml) and m_dry_ is the weight of the membrane after drying (g).

#### 2.3.2. Conductivity

Conductivity was determined by impedance spectrometer with A Zahner–elektrik IM6 (Zahner-elektrik Gmbh, Kronach, Germany) under ambient atmosphere. The impedance was investigated in the frequency range of 200 KHz to 8 MHz at room temperature in a 1 M sulfuric acid solution (amplitude 10 mV). The resistance of membrane was obtained from the intercept of the impedance with the real X-axis. The conductivity was calculated by the following equation.

(2) $$\sigma =\frac{1}{R_{sp}}\;=\frac{d}{R\;\times \;A}\;$$

where σ is the conductivity (mS/cm), R_sp_ is the resistivity (Ω·cm), d is the thickness of membrane (cm), R is the ohmic resistance (Ω) and A is the electrode area (cm^2^).

#### 2.3.3. Water Uptake (WU) and Swelling Ratio (SR)

The dimensional stability such as water uptake and swelling ratio was characterized by comparing the weight, length, width and thickness of a dry and a wetted membrane. An approximately 4 × 1 cm^2^ of wet membrane sample was cut and the weight, length, width and thickness was measured using a balance and a digital Vernier calliper after removal of residual water by wiping out the surface with a tissue paper. After drying the membrane at 90 °C for 1 day, the weight, length, width and thickness were measured again. The water uptake (WU) and swelling ratios (SR—length, width and thickness) were calculated using Equations (3)–(6). Four measurements were taken per membrane from which an average value was obtained.

(3) $$\mathrm{WU}\;\left(\%\right)=\frac{\left(\mathrm{Wet}\;\mathrm{weight}-\mathrm{Dry}\;\mathrm{weight}\right)}{\mathrm{Dry}\;\mathrm{weight}}\times 100\%$$

(4) $$\mathrm{SRL}\;\left(\%\right)=\frac{\left(\mathrm{Wet}\;\mathrm{length}-\mathrm{Dry}\;\mathrm{length}\right))}{\mathrm{Dry}\;\mathrm{length}}\times 100\%$$

(5) $$\mathrm{SRW}\;\left(\%\right)=\frac{\left(\mathrm{Wet}\;\mathrm{width}-\mathrm{Dry}\;\mathrm{width}\right)}{\mathrm{Dry}\;\mathrm{width}}\times 100\%$$

(6) $$\mathrm{SRT}\;\left(\%\right)=\frac{\left(\mathrm{Wet}\;\mathrm{thickness}-\mathrm{Dry}\;\mathrm{thickness}\right)}{\mathrm{Dry}\;\mathrm{thickness}\;}\times 100\%$$

#### 2.3.4. Fourier-Transform Infrared Spectroscopy (FT-IR)

FT-IR spectra of the membranes were recorded by using a Nicolet iS5 (Thermofisher Scientific, Karlsruhe, Germany) and a diamond attenuated total reflectance (ATR) module with 64 scans in the wave number range from 4000 to 400 cm^−1^ under an ambient atmospheric environment.

#### 2.3.5. Gel Content by Extraction

In order to determine the gel content of the membrane, the weight loss was calculated by measuring the weight difference of the dry membranes before and after DMAc extraction. The dry membrane was stored in DMAc for 4 days at 90 °C and the following 3 days in methanol. The weight loss was calculated by using the follow equation.

(7) $$Gel\;\left(\%\right)=\frac{Dry\;weight\;after}{Dry\;weight\;before}\times 100\%$$

#### 2.3.6. Thermal Stability

Thermal stability of the membrane was analysed using a NETZSCH TGA, model STA 499C (NETZSCH, Selb, Germany). Thermal gravimetric analysis (TGA) was performed with a dried membrane (dried at 90 °C in convection oven for 1day before TGA measurement) with a heating rate of 20 °C per minute under an O_2_/N_2_ atmosphere (65–70% oxygen).

#### 2.3.7. Weight Gain Measured in 30% Sulfuric Acid

To determine the weight gain of AEBMs, the dry membranes (sulphated form) were immersed in a 30% sulfuric acid solution at room temperature. The wet weight of the blend membranes was measured after 120 h of doping in the 30% sulfuric acid. The weight gain was calculated using the following equation.

(8) $$Weight\;gain\;\left(\%\right)\;\;\;=\frac{\left(wet\;weight\;-\;dry\;weight\right)\;}{\;dry\;weight}\times \;100\%$$

### 2.4. Vanadium Redox Flow Battery (VRFB) Single Cell Test

A VRFB single cell was assembled as descried in the literature [18]. The anion exchange blend membrane was sandwiched by two carbon felts with an active area of 28 cm^2^ and copper plates as a current collector. The single cell was assembled between two end plates with the screws as a torque of 3.5 Nm. 20 ml of electrolytes (1.6 M vanadium (50% VO^2+^, 50% V^3+^) in 30% H_2_SO_4_) were fed into polyethylene tubes on both sides and allowed to flow through the VRFB without using pumps and tanks. The cell was first charged to 1.6 V and discharged to 1.0 V with the current density of 40 mA/cm^2^, then charged at 40 mA/cm^2^ and discharged at different current densities (six charge and discharge cycles at each current density) for efficiencies evaluation. The open circuit voltage (OCV) was monitored after it was charged to 1.6 V. A long-term charging–discharging cycling test was performed with a same charging and discharging current density of 40 mA/cm^2^. Capacity retention as a function of cycles was calculated by the remaining capacity percent from the beginning capacity. The coulombic efficiency (CE), voltage efficiency (VE) and energy efficiency (EE) for the charging and discharging process were calculated as follows.

(9) $$CE\;\left(\%\right)=\frac{\;t_{d}\;}{t_{c}}\times 100\%$$

(10) $$VE\;\left(\%\right)=\frac{V_{d}}{V_{c}}\times 100\%$$

(11) EE (%)=CE×VE

where t_d_ and t_c_ is the discharging and charging time respectively (s), while V_d_ and V_c_ is the average discharging and charging voltage (V) respectively.

## 3. Results and Discussion

### 3.1. Membranes Preparation

Details of the composition of the AEBMs that were prepared as well as the obtained wet thicknesses are listed in Table 1. PBIs were used as a matrix polymer due to their high thermal and mechanical stabilities [22] enhancing the mechanical stability by forming covalent bonds with the halomethylated polymer. The sulfonated polymers were used as ionical cross-linkers between the sulfonated anions and the imidazolium cations which has previously been shown to enhance the chemical stability of blend membranes [23,24]. Br-PPO was used as the precursor for the quaternization with TMim. The same weight ratios of different combinations of the polymers were used. The variation in the thickness of the blend membranes was probably due to the high viscosity of the fluorinated F6-PBI polymer solutions resulting in the addition of extra solvent, thereby decreasing the concentration of the polymer mixture solution. This resulted in thinner membranes, specifically for the F6-PBI based partially fluorinated polymers-containing blend membranes.

**Figure 3:**
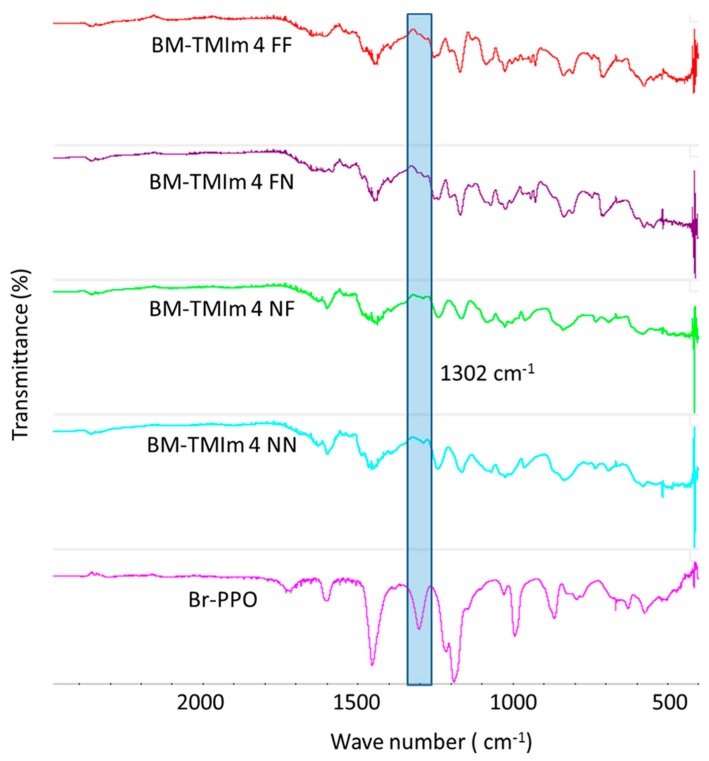
FT-IR spectrum of anion exchange blend membranes and Br-PPO.

To confirm the possible binding of the bromomethyl groups from Br-PPO to the TMIm or PBI, a structural analysis was performed by FT-IR (Figure 3). The strong peak observed at 1302 cm^−1^ can be assigned to a stretching vibration of the CH_2_-Br groups of Br-PPO, which was not observed in the polymers of any of the blend membranes indicating that all CH_2_-Br groups have completely reacted either with TMIm for quaternization or PBI for formation of covalent bonds [18].

### 3.2. Membranes Properties

Various membrane properties of the synthesized AEBMs are summarized in Table 2. Since the same amounts of anion-exchange groups was present in each blend membrane, all the synthesized AEBMs had similar IECs ranging from 2.37 to 2.75 mmol/g. All membranes further also possessed dimensional stabilities that were comparable to those presented in literature [9]. It is known that the anion exchange sites (positively charged) absorb more acid (as HSO_4_^−^ and SO_4_^2−^) when immersed in the electrolytes, which contributes to the ionic conductivity [25]. Therefore higher conductivities of AEMs can be expected than the pure form of AEMs under the acidic conditions in which VRFBs are operated. The conductivities of the AEBMs from this study measured in 1 M sulfuric acid were in the range of 21–27 mS/cm, which was lower than that of Nafion. However, according to the literature, comparable conductivities of AEBMs can be achieved by adjusting the anion exchange polymer ratio [18].

**Figure 4:**
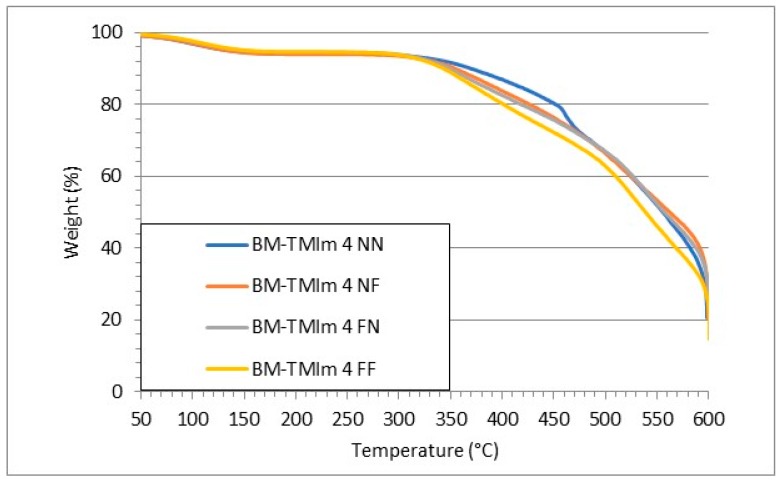
Weight loss as a function of temperature measured by TGA.

To investigate the thermal stability of AEBMs, thermal gravimetric analysis (TGA) was conducted (Figure 4). The first weight loss step (up to 200 °C) is attributed to water evaporation. The second weight loss step starting at approximately 300 °C can be assigned to the splitting-off of the imidazolium groups as well as sulfonated groups from the sulfonated blend component [26]. All AEBMs showed excellent thermal stabilities measured under harsh oxidative conditions (65–70% oxygen). The weight loss measured via solvent extraction showed similar values, compared to our previously published results where more than 90% of the initial membrane weight remained after extraction, indicating successful covalent cross-linking [20].

**Figure 5:**
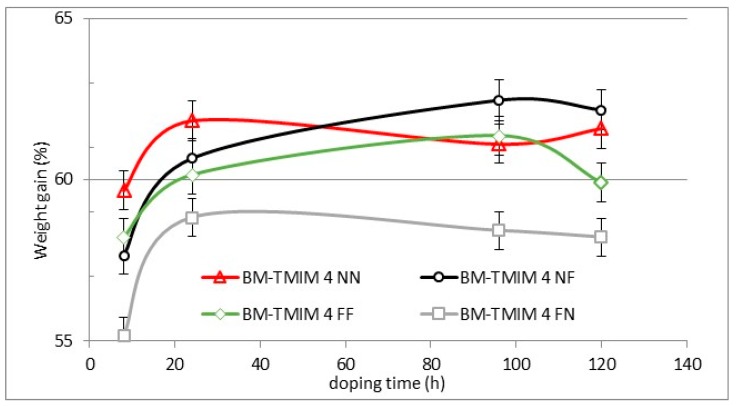
Acid uptake of blend membranes doped in a 30% sulfuric acid solution as a function of time.

The acid uptakes measured in 30% sulfuric acid as a function of immersion time for the blend membranes are shown in Figure 5. All blend membrane showed similar weight gains in a sulfuric acid solution of around 60% after 25 h which then remained near constant for the residual doping time (120 h). Due to the similar IECs of the AEBMs, the weight gain values were expected to be similar for all the investigated AEBMs. From these results complete doping of all the AEBMs can be concluded.

For aromatic backbone based cation exchange membranes, sulfonated Radel chemical degradation was reported via vanadium peroxo radical attack resulting in chain scissions [27]. However, for cross-linked bromomethylated PPO-based AEMs, high chemical stability in vanadium electrolyte solution was reported [28]. Similarly, sulfonic acid doped PBI AEMs showed remarkable stability over 120 days in a 1 M vanadium solution in sulfuric acid at room temperature [29]. The superior chemical stability of AEMs has been attributed to the Donnan repelling effect, leading to limited permeation of vanadium ions into the membranes. In view thereof, a high chemical stability during operation in VRFBs is in principle expected for AEMs. However, in the case of PBI-OO, the electron-rich phenyl ring is prone to sulfonation showing increased peaks intensity in FT-IR spectra in a few days which was doped in 3M sulfuric acid [30,31,32]. As a result, sulfuric acid can be lost via ionical cross-linking as suggested in Figure S1. Thus, FT-IR spectra of F6-PBI membranes was investigated as a function of sulfuric acid doping time in 30% sulfuric acid solution. F6-PBI membrane was doped in sulfuric acid for specific time and de-doped in carbonate solution and membranes were dried. The FT-IR spectra of F6-PBI membranes are shown in Figure S2. No significant changes of spectrum were observed for 9 days doping suggesting that F6-PBI as a matrix polymer is suitable for VRFB.

### 3.3. Vanadium Redox Flow Battery Performance

**Figure 6:**
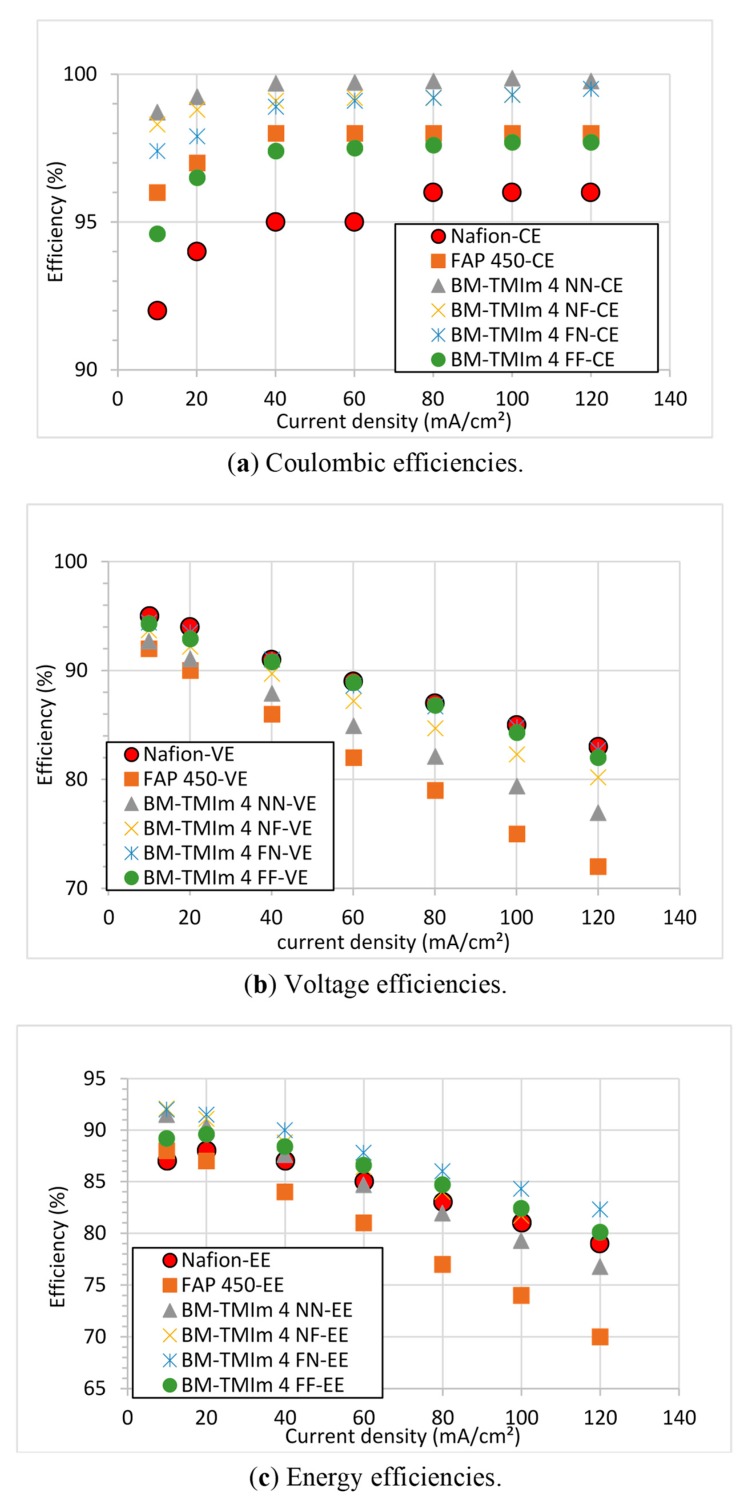
CE (**a**), VE (**b**) and EE (**c**) of studied membranes.

A single cell was used to determine the performance of the AEBMs in a vanadium redox flow battery. The single cell was first charged to 1.6 V to avoid electrode corrosion [32]. Subsequently it was allowed to discharge. Figure 6a,b and c shows the respective CE, VE and EE of the AEBMs, Nafion and FAP 450 membranes as a function of the current density. CE (Figure 6a) is generally affected by the crossover of vanadium ions, side reactions, electrode corrosion as well as by membrane thickness [33]. Since all single cell performances were measured under the same conditions, side reactions and electrode corrosion should be similar for all VRFB experiments. Therefore, the vanadium ion crossover should have the biggest influence on the CE. As expected, all AEBMs showed better CE than Nafion due to the Donnan exclusion effect discussed previously. The CE increased as a function of current density due to the decreased time required for vanadium permeation during charge-discharge at higher current densities [10]. Considering the membrane thickness, the thicker membrane (BM-TMIm 4 NN) showed slightly higher CEs than other AEBMs because of the longer time required for vanadium permeation.

It is known that the VE (Figure 6b) is affected by the membrane’s ohmic resistance corresponding to its conductivity [33]. Nafion showed higher VEs than the AEBMs due to its high conductivity. It can be seen that the VE decreased with increasing current density due to increasing ohmic losses. For AEBMs, even though they have similar conductivities, the thinner membranes (BM-TMIm 4 FF) showed a higher VE than the thicker membranes due to the higher area resistance of the thicker membranes. The AEBMs possessed similar conductivities. Thus thicker membranes have a higher area resistance resulting in lower VEs as was expected [34]. The EE (overall efficiency—Figure 6c) of BM-TMIm 4 FN, BM-TMIm 4 FF and BM-TMIm 4 NF was slightly higher than that of the Nafion membrane due to the higher CEs (VEs were similar) at all current densities. These results confirm that the manufactured AEBMs are promising candidates for VRFBs.

**Figure 7:**
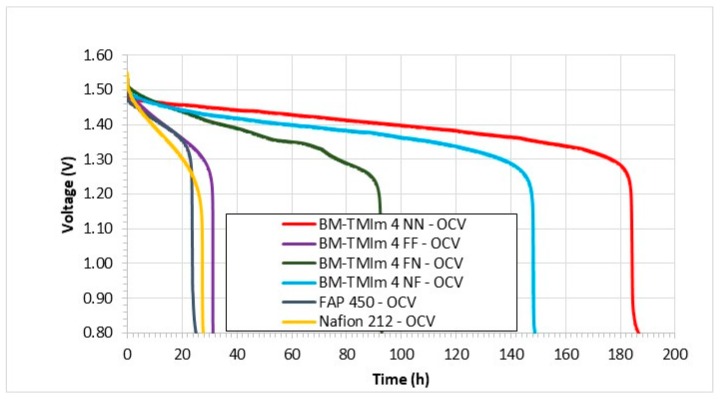
Open circuit voltage (OCV) measurements (self-discharge time) of AEBMs and commercial membranes.

Open circuit voltage (OCV) measurements of VRFBs are considered an indirect way to confirm the rate of the vanadium ion crossover [16]. The OCV of VRFBs was monitored as a function of time after the cell had been charged to 1.6 V (Figure 7). The OCV values gradually decreased as a function of time, followed by a sudden OCV drop with the disappearance of VO_2_^+^ from the positive electrolyte due to vanadium crossover [35]. According to Figure 7, the thinner membrane (BM-TMim 4 FF) showed similar self-discharge times (31 h) as the Nafion membrane (27 h). The self-discharge time of blend membranes increased with increased membrane thickness indicating the correlation between the OCV time and the membrane thickness as would have been expected in view of the influence of membrane thickness on the transport rate of vanadium ions. The thicker membrane (BM-TMIm 4 NN) showed the longest self-discharge time of 184 h. All AEBMs showed longer self-discharge time than commercial membranes even though they are thinner, confirming the Donnan exclusion effect. It should be noted that not all AEMs show longer self-discharge times. If a membrane has poor dimensional stability, the self-discharge time of the membranes shorten [18], confirming that AEMs should be prepared carefully considering their IECs, water uptake values and dimensional stabilities.

**Figure 8:**
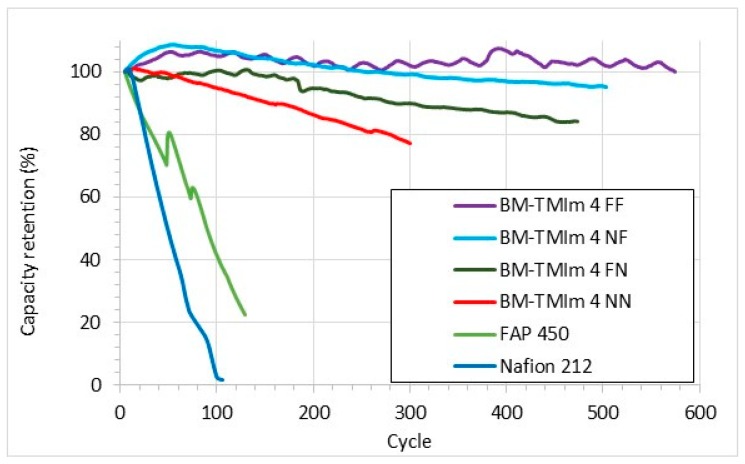
Charge-discharge cycles test run at 40 mA/cm^2^.

To investigate the charge-discharge behaviour of VRFBs, charge-discharge cycles tests of AEBMs were conducted at a current density of 40 mA/cm^2^. The results are shown in Figure 8. Commercial membranes showed fast capacity decay showing respectively 10% and 41% capacity retention for Nafion 212 and FAP 450 after 100 cycles. While the BM-TMIm 4 NN membrane, which had a similar thickness to the commercial membranes, showed a 77% capacity retention after 300 cycles, confirming a high cycling performance. BM-TMIm 4 FF was the most stable membrane with no capacity decay after 550 cycles. According to literature, thicker membranes need higher potentials to achieve the same current density than thinner membranes [34]. This additional potential probably allows vanadium ions to surpass the interface of the membrane and electrode allowing vanadium ions to enter the membrane, resulting in a relatively faster vanadium crossover and capacity decay. Thus, BM-TMIm 4 FF showed excellent capacity retention during the charge-discharge cycles test despite its low thickness. These results once again confirm the suitability of the novel AEBMs presented in this study for VRFB applications.

## 4. Conclusions

Novel anion exchange blend membranes (AEBMs) were prepared and studied in VRFBs. The blend membranes were synthesized with bromomethylated PPO which was quaternized with TMIm. The PBI polymers (non-fluorinated or partially fluorinated) functioned as a matrix polymer for mechanical property enhancement of the AEBMs. The sulfonated polymer (non-fluorinated or partially fluorinated) was used to form ionical cross-links with the 1,2,4,5-tetramethylimidazolium cations of the AEM blend component. All synthesized AEBMs showed similar membrane properties such as IEC, conductivity, thermal stability and dimensional stability. The partially fluorinated polymer-containing blend membranes were chemically stable, as was expected. For the vanadium redox flow battery application, AEMs are known for their high chemical stability due to the Donnan exclusion effect hindering the vanadium ion from entering the membranes thereby limiting the degradation caused by vanadium radical attack. While one problem seemed to be the sulfonation of PBI, resulting in a loss of sulfuric acid during ionical cross-linking, however, F6-PBI as a matrix polymer indicated suitable for VRFB application in acidic conditions showing no structural changes confirmed by FT-IR spectrum. All AEBMs performed better than the commercial membranes (Nafion (a CEM) and FAP 450 (an AEM)) in terms of CE, VE and EE as well as charge-discharge cycles test when used in a VRFB. One of the investigated blend membranes (BM-TMIm4 FF) had excellent capacity retention with no capacity loss during 550 charge-discharge cycles performed at 40 mA/cm^2^. Thus one can conclude that, if the matrix polymer is chosen properly (it has to be thin and stable in acidic environments), the AEBMs are expected to show excellent performance in VRFBs specially, in terms of long term cycling tests. Further studies will focus on the increase of the chemical stabilities of the AEBMs by suitable selection of blend components, particularly for use of anion-exchange blend components having increased chemical stability resulting from both an increase of chemical stability of the polymer backbone and of the cationic group, compared to TMIm-quaternized PPO. A further focus will be to investigate the dependence of the VRFB performance on the thickness of the AEBM.

## Figures and Tables

**Table 1:** Membranes preparation details.

Membrane	Mass of Br-PPO (g)	Mass (g) and Type of PBI	Mass (g) and Type of S-Polymer	Wet Thickness (μm)
BM-TMIm 4 NN	1	1 (PBI-OO)	0.24 (Non-fluorinated polymer)	56 ± 0.96
BM-TMIm 4 NF	1	1 (PBI-OO)	0.24 (Fluorinated polymer)	43 ± 5.25
BM-TMIm 4 FN	1	1 (F6-PBI)	0.24 (Non-fluorinated polymer)	37 ± 0.82
BM-TMIm 4 FF	1	1 (F6-PBI)	0.24 (Fluorinated polymer)	34 ± 0.58
FAP 450	-	-	-	58
Nafion 212	-	-	-	53

**Table 2:** Membrane characterization data.

Membrane	IECs (mmol/g)	Conductivity (mS/cm)	WU (%)	SR_L_ (%)	SR_T_ (%)	SR_W_ (%)	Extraction (%)	T onset (^o^C)
BM-TMIm 4 NN	2.75	21.3 ± 0.8	22 ± 2.0	11 ± 0.3	6 ± 2	11 ± 0.3	93	280
BM-TMIm 4 NF	2.55	25.4 ± 0.8	14 ± 0.9	9 ± 0.7	8 ± 0.7	10 ± 1.0	93	284
BM-TMIm 4 FN	2.58	24.9 ± 0.7	17 ± 1.1	9 ± 0.8	7 ± 0.7	9 ± 1.1	92	278
BM-TMIm 4 FF	2.37	26.6 ± 1.3	13 ± 0.8	8 ± 0.8	4 ± 1.1	7 ± 1.2	92	265
FAP 450	2.18	35.2 ± 7.3	14 ± 3.2	8 ± 0.7	3 ± 1.4	8 ± 1.5	-	305
Nafion 212	0.88 (H^+^)	98.5 ± 5.0	13 ± 1.2	11 ± 0.6	8 ± 1.5	13 ± 2.2	-	300

## Supplementary Materials

The following are available online at http://www.mdpi.com/2077-0375/9/2/31/s1, Figure S1: Possible loss of H_2_SO_4_ during the sulfonation of PBI based blend membranes, Figure S2: FT-IR spectrum of F6-PBI membrane as a function of sulfuric acid doping time (30% sulfuric acid at room temperature).
