# Supplementary material for: Performances of Anion-Exchange Blend Membranes on Vanadium Redox Flow Batteries
Source: Membranes (Basel). 2019 Feb 17;9(2):31. doi: 10.3390/membranes9020031 (PMC6410199; doi:10.3390/membranes9020031)
Supplement: Supplementary file 1 [file membranes-09-00031-s001.pdf]

Supplementary Materials: Performances of Anion-Exchange Blend Membranes on Vanadium Redox Flow Batteries

Hyeongrae Cho [1], Henning M. Krieg [2] and Jochen A. Kerres [1,2,*]

[1] Institute of Chemical Process Engineering, University of Stuttgart, 70199 Stuttgart, Germany; hyeongrae.cho@icvt.uni-stuttgart.de
[2] Faculty of Natural Science, North-West University, Focus Area: Chemical Resource Beneficiation, Potchefstroom 2520, South Africa; henning.krieg@nwu.ac.za

* Correspondence: jochen.kerres@icvt.uni-stuttgart.de; +49-711-68585-244

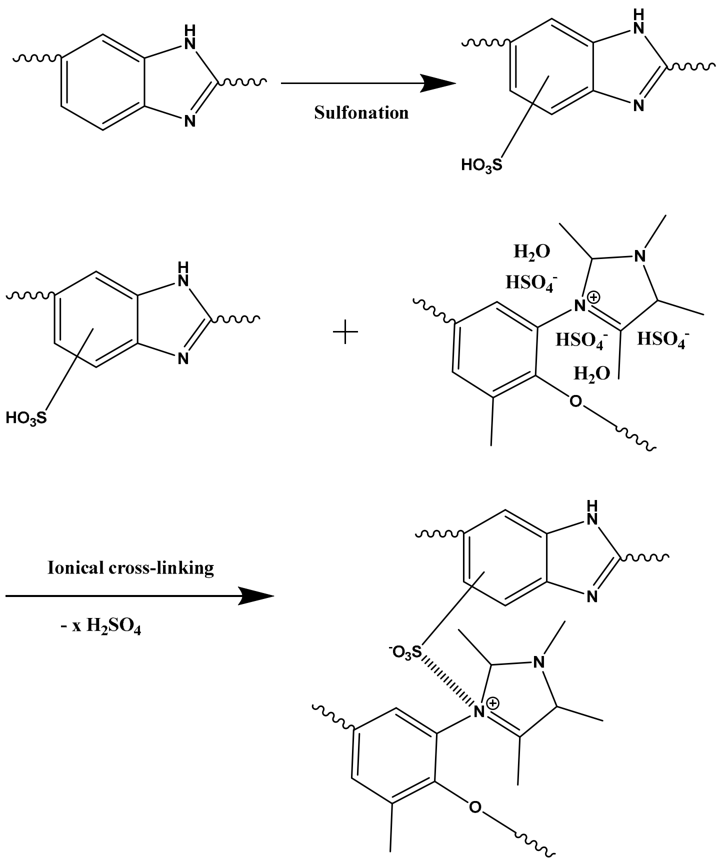

Figure S1. Possible loss of H_2SO_4 during the sulfonation of PBI based blend membranes.

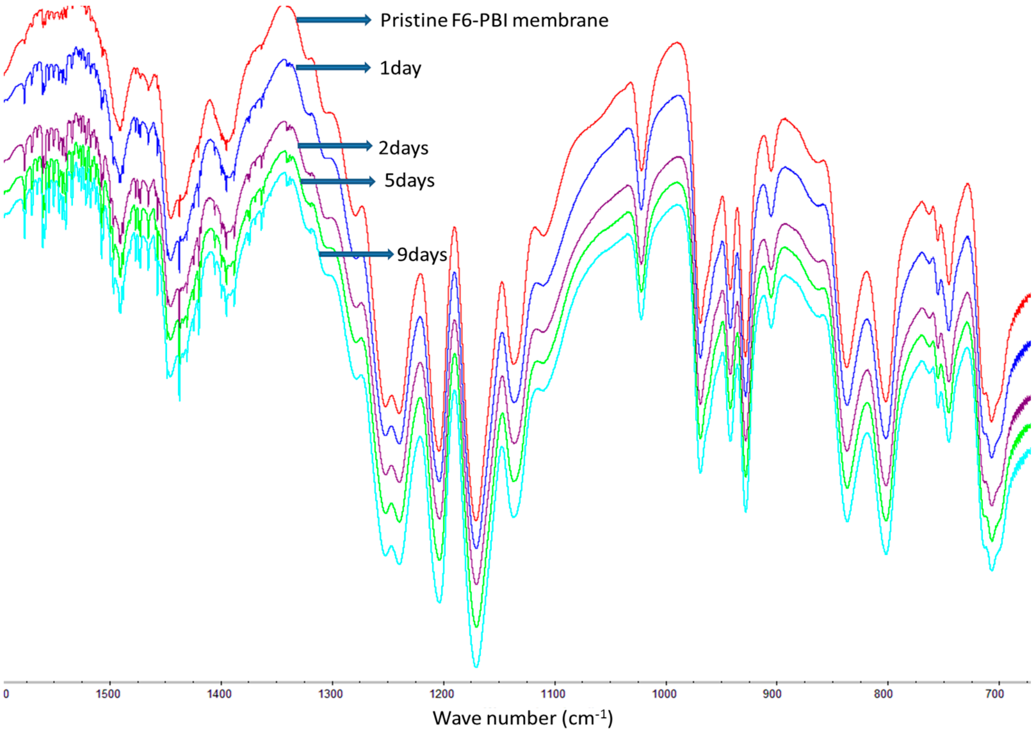

Figure S2. FT-IR spectrum of F6-PBI membrane as a function of sulfuric acid doping time (30% sulfuric acid at room temperature).
